# Machine Learning Based on a Multiparametric and Multiregional Radiomics Signature Predicts Radiotherapeutic Response in Patients with Glioblastoma

**DOI:** 10.1155/2020/1712604

**Published:** 2020-10-24

**Authors:** Zi-Qi Pan, Shu-Jun Zhang, Xiang-Lian Wang, Yu-Xin Jiao, Jian-Jian Qiu

**Affiliations:** Department of Radiation Oncology, Shanghai Huadong Hospital, Fudan University, Shanghai 200040, China

## Abstract

**Methods:**

The MRI images, genetic data, and clinical data of 152 patients with GBM were analyzed. 122 patients from the TCIA dataset (training set: *n* = 82; validation set: *n* = 40) and 30 patients from local hospitals were used as an independent test dataset. Radiomics features were extracted from multiple regions of multiparameter MRI. Kaplan-Meier survival analysis was used to verify the ability of the imaging signature to predict the response of GBM patients to radiotherapy before an operation. Multivariate Cox regression including radiomics signature and preoperative clinical risk factors was used to further improve the ability to predict the overall survival (OS) of individual GBM patients, which was presented in the form of a nomogram.

**Results:**

The radiomics signature was built by eight selected features. The *C*-index of the radiomics signature in the TCIA and independent test cohorts was 0.703 (*P* < 0.001) and 0.757 (*P* = 0.001), respectively. Multivariate Cox regression analysis confirmed that the radiomics signature (HR: 0.290, *P* < 0.001), age (HR: 1.023, *P* = 0.01), and KPS (HR: 0.968, *P* < 0.001) were independent risk factors for OS in GBM patients before surgery. When the radiomics signature and preoperative clinical risk factors were combined, the radiomics nomogram further improved the performance of OS prediction in individual patients (*C*‐index = 0.764 and 0.758 in the TCIA and test cohorts, respectively).

**Conclusion:**

This study developed a radiomics signature that can predict the response of individual GBM patients to radiotherapy and may be a new supplement for precise GBM radiotherapy.

## 1. Introduction

Glioblastoma is the most common malignant tumour of the central nervous system in adults. At present, the standard therapy for glioblastoma patients is surgery and radiotherapy and adjuvant or concurrent chemotherapy [1]. The median survival time of glioblastoma is 14-15 months [2]. However, in the actual clinical practice, the difference in OS of individual GBM patients is very significant [3–5]. As one of the main methods of cancer treatment, radiotherapy plays an important role in the comprehensive multimodal treatment of GBM. In the era of personalized medicine, the core principle of precision medicine is that cancer treatment should be adjusted according to the biological heterogeneity of individual patients. However, the current radiotherapy plan still assumes that each patient benefits from the same dose plan [6], ignoring the heterogeneity of individual tumour patients. It means that clinical practice needs a marker that can predict the response of radiotherapy to lead to more personalized clinical decision making or dose adjustments for patients.

Recent studies have developed and validated some genetic markers for predicting radiotherapeutic response in individual cancer patients. By clustering four different microarray experiments, Kim et al. built a radiotherapeutic response prediction signature [7] containing 31 genes. The 31-gene signature has been validated in independent clinical datasets of different cancer types, including glioblastoma [8], low-grade gliomas [9], head and neck tumours [10], and oesophageal cancer [11]. Eschrich et al. constructed the tumour radiosensitivity index (RSI) [12], which has also been verified in a number of different types of tumour datasets [13].

However, the main drawback of these signatures for predicting response to radiotherapy is that tumour samples must be sequenced, which can only be performed after surgery or biopsy. At the same time, a GBM tissue biopsy is associated with a risk of neurological impairment, and the small samples obtained cannot reflect the overall heterogeneity of the whole tumour.

Therefore, in order to overcome these limitations, it is necessary to develop a noninvasive technology to identify the tumour response to radiotherapy. Radiomics has the advantages of being highly specific and noninvasive. It can mine high-throughput quantitative features from traditional medical images and apply them to clinical decision-making or improve the accuracy of diagnosis and prognosis [14, 15]. Compared with traditional methods, radiomics has two unique advantages. First, radiomics allows semiautomatic or automatic extraction of features and provides more quantitative data than qualitative analysis. Second, by extracting the features of different subregions, the tumour phenotype can be described in depth, which not only reflects the macroscopic characteristics of the tumour tissue but also reflects the molecular characteristics of tumour and the responsiveness to treatment [16–18]. The study by Grossmann et al. showed that extracting the image features of GBM from multiple sequences and multiple subregions can provide a variety of tumour biological information, including information about the cell cycle, inflammation, and immune response, which affects the prognosis of patients [19]. Beig et al. successfully constructed a radiomics scoring model to evaluate hypoxia in GBM patients by using the expression profiles of 21 genes related to the hypoxia pathway of GBM [20]. All of these indicate that radiomics is an extremely promising method to assist in the development of individualized treatment strategies for GBM [21].

This study hypothesized that the radiotherapeutic response of GBM patients may be related to the high-dimensional information in different subregions of MR images and developed a radiomics signature based on a machine learning algorithm to predict the radiotherapeutic response of GBM patients. The performance for predicting the OS of individual GBM patients was further improved by constructing a nomogram combining the imaging markers and clinical factors.

## 2. Methods and Materials

### 2.1. Patients

In this retrospective study, a total of 152 pathologically confirmed GBM patients were included: 122 from the TCGA-GBM [22] dataset in the cancer imaging database (TCIA) [23] and 30 from a local hospital dataset (January 2013 to February 2019). To evaluate the prognostic value of radiomics signature, OS was calculated as the time from the initial diagnosis to death or censure point (June 15, 2020) if patients were still alive. At the median 14.7-month follow-up, 11 (36.67%) patients from the local hospital were alive.

TCIA (http://www.cancerimagingarchive.net) is a publicly available database, and the medical images of patients are deidentified; therefore, it does not need the approval of the institutional review committee. The data from the local hospital were approved by the ethics committee of the hospital, and informed consent of the patients was waived. All images were obtained at the time of the initial diagnosis.

The inclusion criteria for the TCIA dataset used to build the radiomics signature were as follows: (1) newly diagnosed histologically confirmed GBM (WHO classification IV); (2) preoperative MRI images with a complete sequence, including T1-weighted, postcontrast T1-weighted, T2-weighted, and T2 flair (T1W, T1c, T2W, and T2FLAIR, respectively) images; (3) original dataset with corresponding gene expression values (HU-133A); and (4) satisfactory image quality.

**Figure 1:**
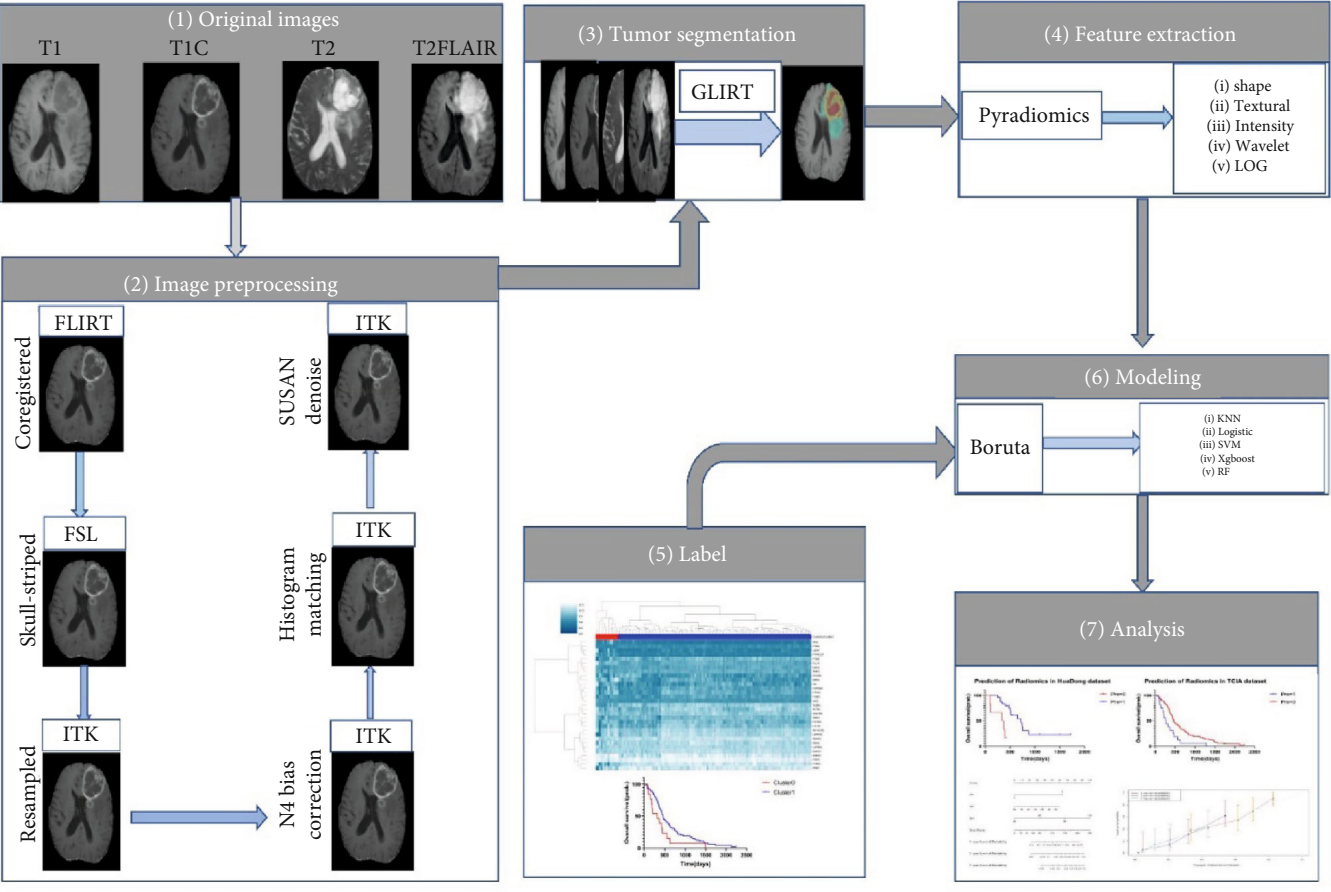
A flowchart describing the radiomics method for prediction of radiotherapy response. (1) Preprocess the original image; (2) delineate the subregion ROI by GLISTR; (3) feature extraction using pyradiomics; (4) 31-gene signature were used to predict the result of the corresponding data, and a label was generated. (5) Feature selection by the Boruta algorithm; (6) modeling by a variety of machine learning methods, ROC curve, and AUC evaluation model; (7) building a prediction model by combining radiomics signature features and clinical features, finally displaying the OS prediction results by nomogram.

Using patients from a local hospital (*n* = 30) and the TCIA dataset receiving radiotherapy (*n* = 102), the ability of the radiomics signature to predict individual GBM patients' radiotherapeutic responses was verified. The inclusion criteria for these data were as follows: (1) GBM (WHO classification IV) with newly diagnosed histology; (2) postoperative radiotherapy; (3) preoperative MRI images with complete sequences, including T1-weighted, postcontrast T1-weighted, T2-weighted, and T2 flair (T1W, T1c, T2W, and T2FLAIR, respectively); (4) satisfactory image quality; and (5) OS that could be achieved through follow-up. The flowchart of this study is shown in Figure 1. The detailed data exclusion process in TCIA is described in Supplement S1 and Figure S1, and the local dataset is described in Supplement S2 and Figure S2.

### 2.2. MRI Data Acquisition

#### 2.2.1. MR Image Acquisition of TCIA Cohort

MRI was performed with a 1.5 or 3.0 T scanner before operation. In TCIA images, the T1 sequence parameters were as follows: TR/TE, 352-3379 msec/2.75-19 msec; and slice thickness, 1-5 mm. The parameters of the T1 enhancement sequence were as follows: repeat time (TR)/echo time (TE), 4.9–3285 msec/2.1–20 msec and slice thickness, 1–5 mm. The parameters of the T2 sequence were as follows: TR/TE, 700-6370 msec/15-120 msec and slice thickness, 1.5-5 mm. The parameters of the T2FLAIR sequence were as follows: TR/TE, 6000–11000 msec/34.6–155 msec and slice thickness, 2.5–5 mm.

#### 2.2.2. Local Cohort MR Image Acquisition

Preoperative MRI was performed with a 3.0 T scanner (GE Signa HD xt) and 8-channel array coil. In the images from the local hospital, the parameters of the T1 sequence were as follows: TR/TE, 139-409 msec/2.46-2.48 msec and slice thickness, 5 mm. The parameters of the T1 enhancement sequence were as follows: TR/TE, 220–2300 msec/2.34–2.5 msec and slice thickness, 1-5 mm. The parameters of the T2 sequence were as follows: TR/TE, 4000-6000 msec/92-125 msec and slice thickness, 5 mm. The parameters of the T2FLAIR sequence were as follows: TR/TE, 7000–9000 msec/81–85 msec and slice thickness, 5 mm.

### 2.3. Radiotherapeutic Response

The TCGA-GBM dataset in the TCGA (The Cancer Genome Atlas) database was used to evaluate the radiotherapeutic response of individual GBM patients. Gene expression data (HU-133A microarray) corresponding to the TCIA image data were obtained from the UCSC Xena browser (https://xenabrowser.net). According to the previous study [7] of Kim et al., a 31-gene model was used to evaluate the radiotherapeutic response, which is a model to calculate the SF2 (2 Gy survival fraction, which represented radiosensitivity) value distribution of individual patients through gene expression.

Based on the expression of the specific 31 genes in the TCGA dataset, the patients were divided into two groups by a hierarchical clustering method (*k* = 2): radiotherapy effective group (RE) and radiotherapy resistance group (RR). Kaplan-Meier survival analysis was performed to verify the prediction results of the 31-gene model.

### 2.4. Image Preprocessing and Tumour Segmentation

Image preprocessing was mainly performed through the FMRIB software library (http://fsl.fmrib.ox.ac.uk/fsl/fslwiki/FSL) and with Python's SimpleITK package. To increase the robustness of features as much as possible through preprocessing [24], the following steps were adopted in this study: use of FLIRT in FMRIB to coregister the same T1WI image [25] as the template. After skull stripping, the isotropic voxel was resampled [26] to 1 × 1 × 1 mm^3^. N4ITK [27] was used to correct the bias field of each image sequence to eliminate the influence of pixel extremum in the image as much as possible. Since the image data were collected by different centres, a landmark-based method [28] was used to standardize the intensity. Then, the SUSAN method [29] (Smallest Univalue Segment Assimilating Nucleus) was used to smooth the image to reduce the interference of high-frequency intensity changes in different images. The image preprocessing process is shown in Figure 1.

**Figure 2:**
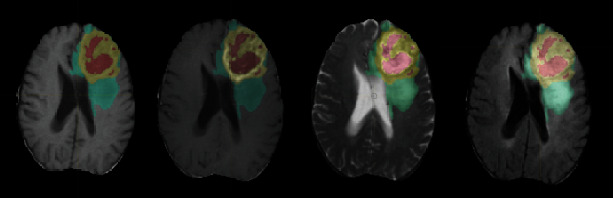
The segmentation result of tumour subregions overlapped on T1WI, CE-T1WI, T2WI, and FLAIR images.

GLISTR (glioma image segmentation and registration) software [30] was used to segment the image automatically. After preprocessing, the image was automatically divided into four segmentation subregions, i.e., the tumour enhancement area, tumour nonenhancement area, peritumoural edema area, and whole tumour. After that, two different radiotherapy doctors reviewed and revised the segmentation results together.  Figure 2 shows an example of the segmentation results.

### 2.5. Radiomics Feature Extraction

Based on the above four subregions, five groups of features were extracted by pyradiomics including the following: (I) shape features, (II) intensity features, (III) texture features, (IV) intensity and texture features under wavelet transformation, and (V) intensity and texture features under Gaussian Laplace transformation. Shape features describe the shape and volume of the tumour. Intensity features refer to the first-order statistics of all voxel intensity values in the region of interest (ROI). Texture features use the Gray Level Cooccurrence Matrix (GLCM), Gray Level Dependence Matrix (GLDM), Gray Level Run-Length Matrix (GLRLM), Gray Level Size-Zone Matrix (GLSZM), and Neighbouring Gray Tone Difference Matrix (NGTDM) to quantify sharp changes in the gray spectrum. Wavelet and Gaussian Laplacian features are obtained by extracting voxel features and texture features, respectively, after applying wavelet or Gaussian Laplace transformation to the image. These two processes can obviously show the features of the image edge. Finally, for each image, 28496 features were extracted from four segmentation regions of four sequences. For the detailed definition of features, please refer to Supplement S3.

### 2.6. Radiomics Feature Selection

The Boruta algorithm [31] was used for feature selection. Boruta is a packing algorithm for selecting all relevant features. After comparing the importance of original features and random features for modeling, the important features were arranged from top to bottom, and the *P* values were corrected by the Benjamin Hochberg method to ensure their reliability. To ensure the robustness and replicability of features, 20 cases were randomly selected from all datasets. A region of interest (ROI) was generated after GLISTR automatic segmentation. The generated ROI was modified by different doctors to generate two independent groups of new ROIs.

The two new groups of new ROIs including four subregions were used to extract the corresponding radiomics features from four sequences of MRI images in 20 cases, and the intraclass correlation coefficient (ICC) of each feature was calculated [32]. Among them, the features with an ICC of 0.9 were considered to be robust [33] and were included in the study.

### 2.7. Radiomics Signature and Nomogram Construction

To develop the radiomics signature, all cases from the TCIA database were stratified and sampled by a computer-generated random number according to the ratio of 2 : 1 and were divided into a training set (*n* = 82) and a validation set (*n* = 40). Because the data from the RE and RR groups were unbalanced, the training set was first balanced by using the synthetic minority oversampling technique (SMOTE) algorithm [34]. The SMOTE algorithm is a kind of oversampling algorithm for fewer classes. It is generally considered that it can effectively balance imbalanced samples. Several machine learning algorithms, such as logistic regression (logistic), random forest (RF), support vector machine (SVM), *k*-nearest neighbour (KNN), and Xgboost (Xgboost, extreme gradient boosting), were used to model. The input of the model was the selected features, and the output was the results of the 31-gene model prediction.

The purpose of using these machine learning methods was to build a model to predict the radiotherapeutic response of GBM patients by inputting quantitative image features before surgery. Through 10-fold cross-validation, a grid search was carried out on the training set to determine the optimal tuning parameters of each machine learning algorithm. The AUC and ROC curve were used to evaluate the model in the training set and validation set, respectively, and the most suitable model was selected.

Due to the lack of gene expression data in local hospitals, we used an indirect method to verify the model independently. We hypothesized that among the 30 local GBM patients with basically the same treatment, the OS of the cluster with strong response to radiotherapy should be longer than that of the patients with radiotherapy resistance and was verified by Kaplan survival analysis. In order to further construct the OS prediction model for individual GBM patients, univariate and multivariate Cox regression models were used to evaluate the effects of the radiomics signature and clinical factors (such as KPS, age, gender, and tumour location) on OS. Multivariate Cox regression was performed for independent risk factors and presented in the form of a nomogram. The calibration curve was used to evaluate the consistency between the nomogram and the observed values, and the Harrell consistency index (*C*-index) was used to quantify the discrimination performance.

### 2.8. Statistical Analysis

All data were analyzed by SPSS (version 19.0), R software (3.4), and Python (3.7). Pearson's chi-squared test or Student's *t*-test (as appropriate) were performed with SPSS to evaluate the differences between the TCIA and local hospital datasets in terms of age, gender, KPS, survival status, OS, etc. The statistical significance levels were all two-sided, with the statistical significance level set at 0.05. The *C*-index was calculated with the “hmisc” software package. ROC curves were drawn using the “pROC” package. Feature selection used the R package which is “Boruta,” and classifier building was mainly performed using the following Python packages: “Gridsearchcv,” “Sklearn,” “SMOTE,” and “Xgboost.”

## 3. Results

### 3.1. Patient Characteristics

**Figure 3:**
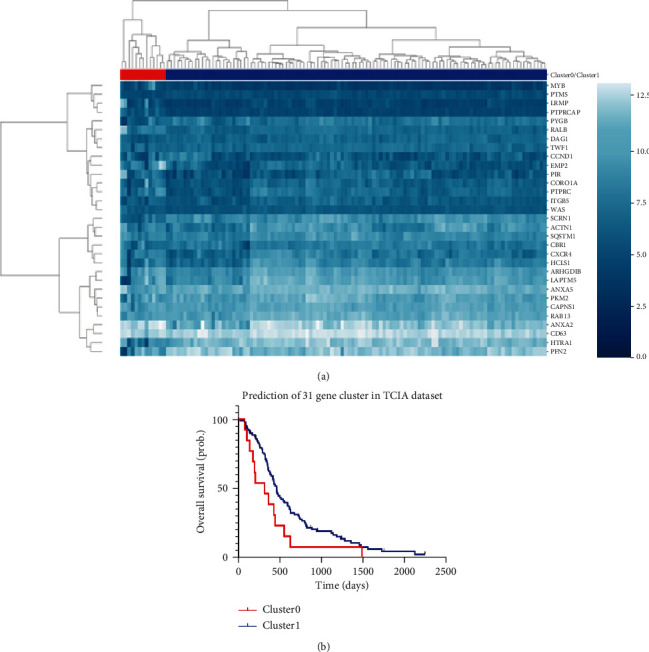
Hierarchical clustering was used to determine the expression pattern of the 31-gene signature on the samples from TCGA (Figure 3(a)). The samples in the red branch on the left side of the dendrogram are classified as Cluster0, while the samples in the blue branch on the right side are classified as Cluster1. After using Kaplan-Meier survival analysis, the prognosis of Cluster0 is different from that of Cluster1, so according to Kim et al.'s report, Cluster0 was subclassified as the radiotherapy resistance group (RR), whereas Cluster1 was a radiotherapy effective group (RE) (Figure 3(b)).

First, gene expression data of 122 patients were included in the 31-gene model to calculate the distribution of the SF2 value. In order to test the accuracy of this distribution, Kaplan survival analysis was performed in 102 patients who received radiotherapy. It can be seen that Cluster0 in the red part on the left side is the RR group, and Cluster1 in the blue part is the RE group (*P* < 0.05), as shown in Figure 3.

The clinical data and results of the model are summarized in Table 1. There was no significant difference in age, gender, KPS, and OS between the TCIA dataset and independent test group (*P* > 0.05). In this study, 122 cases from the TCIA dataset were divided into the RR group (13 cases) and the RE group (109 cases). The reason for this result is that the 31-gene model is a marker to distinguish the responsiveness to radiotherapy in the sample, and the results of the cluster analysis depend on the sample size and median value.

### 3.2. Feature Selection

After using the Boruta algorithm for feature selection and applying the Benjamin Hochberg method to correct the *P* value, 8 features were retained, as shown in Table 2.

All features selected by the Boruta algorithm were qualified (the ICC value was higher than 0.9). A summary of the ICC results for the features can be found in Supplement S4 and Figure S3.

### 3.3. Radiomics Signature Construction

**Figure 4:**
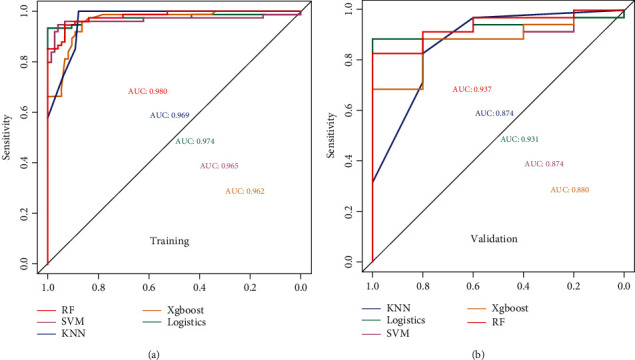
Receiver operating characteristic (ROC) curves of training (a) and validation (b) sets under different machine learning algorithms. It can be seen from the figure that the random forest algorithm performs best. KNN: *k*-nearest neighbour; logistics: logistic regression; SVM: support vector machine; Xgboost: extreme gradient boosting; RF: random forest.

The AUCs of RF, SVM, KNN, logistic, and Xgboost were 0.980, 0.965, 0.969, 0.974, and 0.962, respectively, and 0.937, 0.874, 0.874, 0.931, and 0.880, respectively. The ROC curves of the five machine learning methods are shown in Figure 4, and the accuracy, sensitivity, and specificity of the model are summarized in Tables 3 and 4. Because of the best performance, the RF model was chosen as the final radiomics signature model.

### 3.4. Survival Analysis

Individual GBM patients' radiotherapeutic responses to radiotherapy in the TCIA (*n* = 102) and test (*n* = 30) datasets were predicted using radiomics signature.

**Figure 5:**
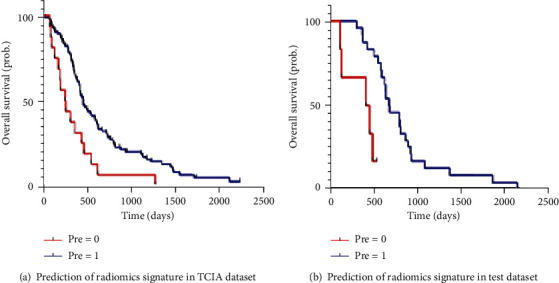
Using Kaplan-Meier analysis to verify the performance of radiomics signature. The response ability of GBM patients to radiotherapy was successfully divided into the high-risk group (radiotherapy resistance group, pre = 0) and low-risk group (radiotherapy effective group, pre = 1) according to the prediction results of radiomics signature. There were significant differences in TCIA (a) and test (b) datasets between the high-risk group and the low-risk group.

After Kaplan-Meier analysis, as shown in Figure 5, the *C*-index of the radiomics signature in the TCIA and test datasets was 0.703 (95% CI: 0.642-0.764, *P* < 0.001) and 0.757 (95% CI: 0.663-0.851, *P* = 0.001), respectively.

### 3.5. Construction and Evaluation of Nomogram

Univariate and multivariate Cox regression analyses using the radiomics signature, age, and KPS as independent risk factors were performed (radiomics signature: HR: 0.290, 95% CI: 0.160-0.526, *P* < 0.001; age: HR: 1.023, 95% CI: 1.005-1.040, *P* = 0.01; and KPS: HR:0.968, 95% CI: 0.950-0.987, *P* < 0.001).

**Figure 6:**
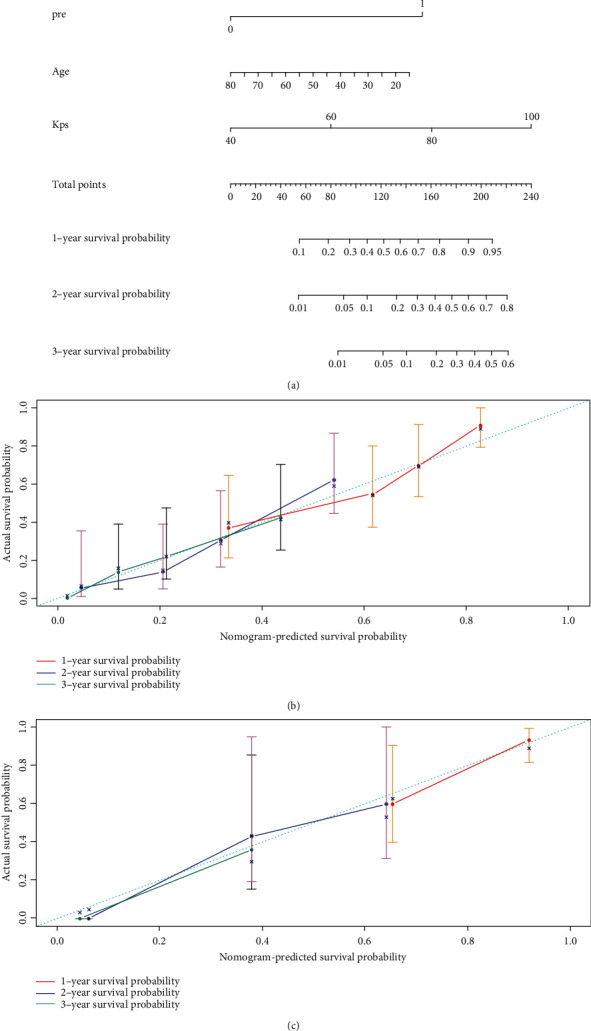
The nomogram constructed by radiomics signature, KPS, and age (a). The calibration curve of the nomogram shows that the prediction and observation possibilities of TCIA (b) and test (c) datasets are satisfactory.

According to the relevant factors of the multivariate Cox regression analysis, the nomogram was constructed (Figure 5). The *C*-index of the nomogram in the TCIA dataset was 0.764 (95% CI: 0.723-0.806, *P* < 0.001), and that of the test dataset was 0.758 (95% CI: 0.667-0.838, *P* < 0.001), indicating that the prediction performance was improved. The calibration curves of 1-, 2-, or 3-year OS probability after radiotherapy are shown in Figure 6. The calibration curve of the nomogram shows that there is satisfactory consistency between the prediction and observation possibility of OS in 1, 2, and 3 years in the TCIA and independent test datasets.

## 4. Discussion

This study built a radiomics signature based on three texture, one shape, and four intensity features and verified that it can predict the response of individual patients to radiotherapy on an independent test dataset.

Since the model constructed in this study only predicted the results of a 31-gene model through preoperative images, it was not necessary to consider whether the patient had received radiotherapy in the modeling stage of this study. In the validation stage of this model, because the 31-gene model is only a model with predictive effect in patients who have received radiotherapy, only 102 of 122 patients who have received radiotherapy are included in the validation phase.

Different from other studies using radiomics to predict the response to radiotherapy [35], this study used a 31-gene signature. This is because there are many confounding factors in reflecting the response ability of patients to radiotherapy with clinical results. However, the 31-gene clustering model based on the SF2 value can only predict the individual's response ability after radiotherapy [7] and has been verified [8]. Therefore, the radiomics signature constructed in this study can be verified by a Kaplan-Meier survival curve; that is, the OS of the sensitive cluster is longer than that of the resistant cluster.

Since the model constructed in this study only predicted the results calculated by a 31-gene model through preoperative images, it was not necessary to consider whether the patient had received radiotherapy in the modeling stage of this study. In the validation stage of this model, because the 31-gene model is only a model with predictive effect in patients who have received radiotherapy, only 102 of 122 patients who have received radiotherapy are included in the validation phase.

In order to further improve the ability to predict the OS of individual patients, this study constructed a nomogram including the radiomics signature and preoperative clinical factors. The *C*-index of the nomogram was 0.764 (95% CI: 0.723-0.806, *P* < 0.001) and 0.758 (95% CI: 0.667-0.838, *P* < 0.001) in the TCIA dataset and independent test dataset, which was higher than that with the single application of the radiomics signature (the *C*-index of the TCIA dataset was 0.703, 95% CI: 0.642-0.764, *P* < 0.001; and the *C*-index of the independent test dataset was 0.757, 95% CI: 0.663-0.851, *P* = 0.001), indicating that the combination of multiple risk factors can improve the ability to predict the OS of individual GBM patients. MGMT methylation or IDH mutation status was not included in this study because the purpose of this study was to build a radiomics signature to predict the response of individual GBM patients to radiotherapy by extracting preoperative imaging features. MGMT methylation and IDH mutation status need to be obtained after operation or biopsy, which undoubtedly limits the application of the nomogram in patients who cannot be operated.

Multiparameter imaging sequences contain the comprehensive information of the tumour; for instance, T1WI images reflect the anatomical information of the tumour, and CE-T1WI images include information regarding tumour local angiogenesis and blood-brain barrier damage. Previous studies have shown that multisequence imaging features can be used to predict the heterogeneity and gene expression of individual tumours. The radiomics signature included 1 edema subregion, 4 tumour enhancement subregions, 1 tumour nonenhancement subregion, and 2 overall tumour features. This may be because the nonenhancement subregion is related to the process of apoptosis. The features of the enhancement subregion are related to the process of signal transduction and protein folding, and the edema subregion mainly reflects the process of the cell cycle [19]. These tumour biological pathways are related to the function of the genes in the 31-gene signature and have been proven to be related to the response ability of cells to radiation [7].

The core of precision medicine is to make clinical decisions according to individual heterogeneity. For GBM, radiotherapy has become an important part of standard therapy. How to choose the most appropriate treatment strategy or adjust the radiotherapy dose parameters to better match the biological phenotypes of individual patients has become a problem.

In a recent study [36], the authors combined gene expression values with traditional LQ models to build a dose-response model for individual patients. According to the model, patients can be divided into several clusters, and the response degree of each cluster to the same dose is very different. This shows that it is necessary to adjust the dose of radiotherapy for individual patients. At present, the recommended standard procedure postoperative treatment is the combination of conventional fractionated radiotherapy (RT) and temozolomide (TMZ), followed by adjuvant TMZ [37]. Neoadjuvant TMZ can improve the sensitivity of patients to radiotherapy [37], and a phase II clinical trial has confirmed that the use of neoadjuvant TMZ before radiotherapy can increase the OS of GBM patients [38]. Tumour-treating fields (TTFs) is the latest GBM recommended therapy in the NCCN (National Comprehensive Cancer Network) guidelines, and a previous clinical study found that in addition to its antimitotic effect, this technique can also specifically delay DNA repair and increase DNA-induced damage, thus increasing the radiosensitivity of tumour cells [39–40]. Therefore, it is very important to predict the response of GBM patients to radiotherapy. For those clusters with radiotherapy resistance, the individual clinical decision of using TTF or neoadjuvant TMZ before radiotherapy may prolong the OS of individual patients.

There are some limitations to this study. First, although the study included an independent test dataset, it had a relatively small sample size with retrospective data. Increasing the sample size to improve the robustness of the model is the main work in the next stage. To ensure the robustness and repeatability of the research, multicentre data should be collected. Second, limited by the TCIA database, only four kinds of conventional MRI sequences (T1WI, CE-T1WI, T2WI, and T2FLAIR) were used in the dataset in this study, but no other sequences (such as DCE or DTI) were included. Third, a more accurate OS should be from the time of radiotherapy to death or censure point. Although postoperative radiotherapy has been the standard treatment for GBM, due to the limitations of the TCIA dataset, detailed treatment information (such as the use and type of chemotherapy drugs, the dose of radiotherapy, or the start time of radiotherapy) cannot be obtained. Therefore, this study used the time from diagnosis to censure point as OS to roughly evaluate the impact of radiotherapy on survival. This rough evaluation has a certain impact on the accuracy of this study. Finally, although a variety of image preprocessing methods are used in this study, different imaging parameters and protocols still affect its radiologic characteristics to a certain extent. Moreover, most of the image parameters are removed from TCIA image data, so the normalization method cannot be used further. This is the main reason why the application of radiomics is currently limited.

## 5. Conclusion

In this study, a noninvasive radiomics signature was built by combining the previous 31-gene signature with radiomics, which was proven to predict the response of GBM patients to radiotherapy on independent test datasets. Compared with the 31-gene model prediction after surgery, the radiomics signature constructed by the machine learning algorithm can predict the response ability of radiotherapy before operation. The performance of predicting individual patients' OS can be further improved by using the constructed nomogram with the radiomics signature, age, and KPS, and this technique may be a new attempt for providing precise GBM radiotherapy.

## Figures and Tables

**Table 1:** Characteristics of patients in the TCIA and independent test datasets.

Characteristic	TCIA (*N* = 102)	Huadong (*N* = 30)	*P*
Ages (years)			0.856
Range	17-80	18-73	
Median	57.5	54	
Mean ± SD	56.10 ± 14.35	52 ± 13.68	
Gender, No. (%)			0.847
Female	40 (39.22%)	13 (43.33%)	
Male	62 (60.78%)	17 (56.67%)	
Status, No. (%)			0.0011
Alive	10 (9.8%)	11 (36.67%)	
Dead	92 (90.2%)	19 (63.33%)	
KPS			0.2795
KPS > 60	75	21	
KPS ≤ 60	17	9	
Not reported	10	0	
Tumour location			0.377
Frontal lobe	24	12	
Temporal lobe	43	11	
Parietal lobe	19	4	
Occipital lobe	8	3	
Insular lobe	6	0	
Callosum lobe	2	0	
OS (months)			0.6516
Range	1-74.87	3.3-52.43	
Median	14.30	14.77	
Mean ± SD	18.90 ± 15.23	17.53 ± 10.70	
31-gene prediction result			
RH	89	—	
RR	13	—	
Radiomics prediction result			0.8813
RH	85	24	
RR	17	6	

**Table 2:** A summary of the high-throughput radiomics features extracted.

MRI sequences	Region	Group	Feature name	Type
T1WI	Whole tumour	Shape	MinorAxisLength	Origin
CE-T1WI	Edema	Texture	MeanAbsoluteDeviation	Wavelet-HHL
CE-T1WI	Enhancement	Texture	GLCM_JointEnergy	Wavelet-LLH
CE-T1WI	Enhancement	Texture	GLDM_DependenceNonUniformityNormalized	Wavelet-LLH
CE-T1WI	Enhancement	Intensity	90Percentile	Wavelet-LHH
CE-T1WI	Enhancement	Intensity	90Percentile	Wavelet-HLH
T2WI	Whole tumour	Intensity	90Percentile	Log-sigma-1-mm
T2WI	Nonenhancement	Texture	GLSZM-SizeZoneNonUniformity	Wavelet-LHH

**Table 3:** A summary of the AUC with different machine learning methods on the TCIA dataset.

Algorithm	AUC	95% CI	SENS	SPEC	ACC	*P*
RF	0.980	0.942-0.996	0.946	0.932	0.939	<2.2*e*-16
SVM	0.965	0.921-0.988	0.905	0.972	0.939	<2.2*e*-16
KNN	0.969	0.926-0.990	0.757	0.932	0.844	<2.2*e*-16
Logistic	0.974	0.933-0.993	0.932	1	0.966	<2.2*e*-16
Xgboost	0.962	0.917-0.987	0.865	0.905	0.885	<2.2*e*-16

SENS: sensitivity; SPEC: specificity; ACC: accuracy.

**Table 4:** A summary of the AUC with different machine learning methods on the independent test dataset.

Algorithm	AUC	95% CI	SENS	SPEC	ACC	*P* value
RF	0.937	0.813-0.989	0.829	1	0.9	<2.2*e*-16
SVM	0.874	0.732-0.957	0.771	0.800	0.775	1.375*e*-08
KNN	0.874	0.731-0.958	0.714	0.800	0.725	7.63*e*-05
Logistic	0.931	0.805-0.987	0.886	1	0.900	<2.2*e*-16
Xgboost	0.880	0.738-0.961	0.886	0.800	0.875	6.851*e*-09

SENS: sensitivity; SPEC: specificity; ACC: accuracy.

## Data Availability

The data from TCIA and UCSC can be downloaded from http://www.cancerimagingarchive.net and https://xenabrowser.net. The data from the local hospital can be obtained from the corresponding author as reasonably required.

## Supplementary Materials

Supplementary Materials Supplements S1 and S2 provide a text description of the screening criteria for samples from TCIA and local datasets and are accompanied by two flowcharts, Figures S1 and S2. These two parts are mentioned in Section 2.1 of the paper. Supplement S3 describes the definition and details of five groups of radiomics features, which are mentioned in Section 2.5 of the paper. Supplement 4 describes the robustness test by calculating the ICC values of the radiomics features and presenting the results in the form of graphs (Figure S3). This part is mentioned in Section 3.2 of the original text.
