# Supplementary material for: Machine Learning Based on a Multiparametric and Multiregional Radiomics Signature Predicts Radiotherapeutic Response in Patients with Glioblastoma
Source: Behav Neurol. 2020 Oct 24;2020:1712604. doi: 10.1155/2020/1712604 (PMC7604589; doi:10.1155/2020/1712604)
Supplement: Supplementary Materials — Supplements S1 and S2 provide a text description of the screening criteria for samples from TCIA and local datasets and are accompanied by two flowcharts, Figures S1 and S2. These two parts are mentioned in Section 2.1 of the paper. Supplement S3 describes the definition and details of five groups of radiomics features, which are mentioned in Section 2.5 of the paper. Supplement 4 describes the robustness test by calculating the ICC values of the radiomics features and presenting the results in the form of graphs (Figure S3). This part is mentioned in Section 3.2 of the original text. [file 1712604.f1.docx]

**Supplement S1. Inclusion and Exclusion Criteria of TCIA**

**According to the inclusion criteria presented (Fig. S1), a total of 262 patients i) from the Cancer Genome Atlas (TCGA) GBM Project and ii) their corresponding MRI data from the Cancer Imaging Archive (TCIA) were retrospectively included. Then, 157 patients were excluded for the following reasons: i) lack of at least one of the following MRI sequences from TCIA: T1-weighted gadolinium contrast-enhanced, T1-weighted, T2-weighted, and T2-weighted FLAIR sequences (T1C, T1, T2, FLAIR) (n=107); ii) the MRI sequences were acquired after surgery or biopsy (n=20). iii) the MRI sequences were acquired with severe motion or artifacts that may have induced bias in subsequent analyses (n=13).In total, 122 patients were eligible for this study. According to the prediction results of 31gene model, these patients were divided into Training set (n = 82) and test set (n = 40) after 2:1 random stratified sampling**

**
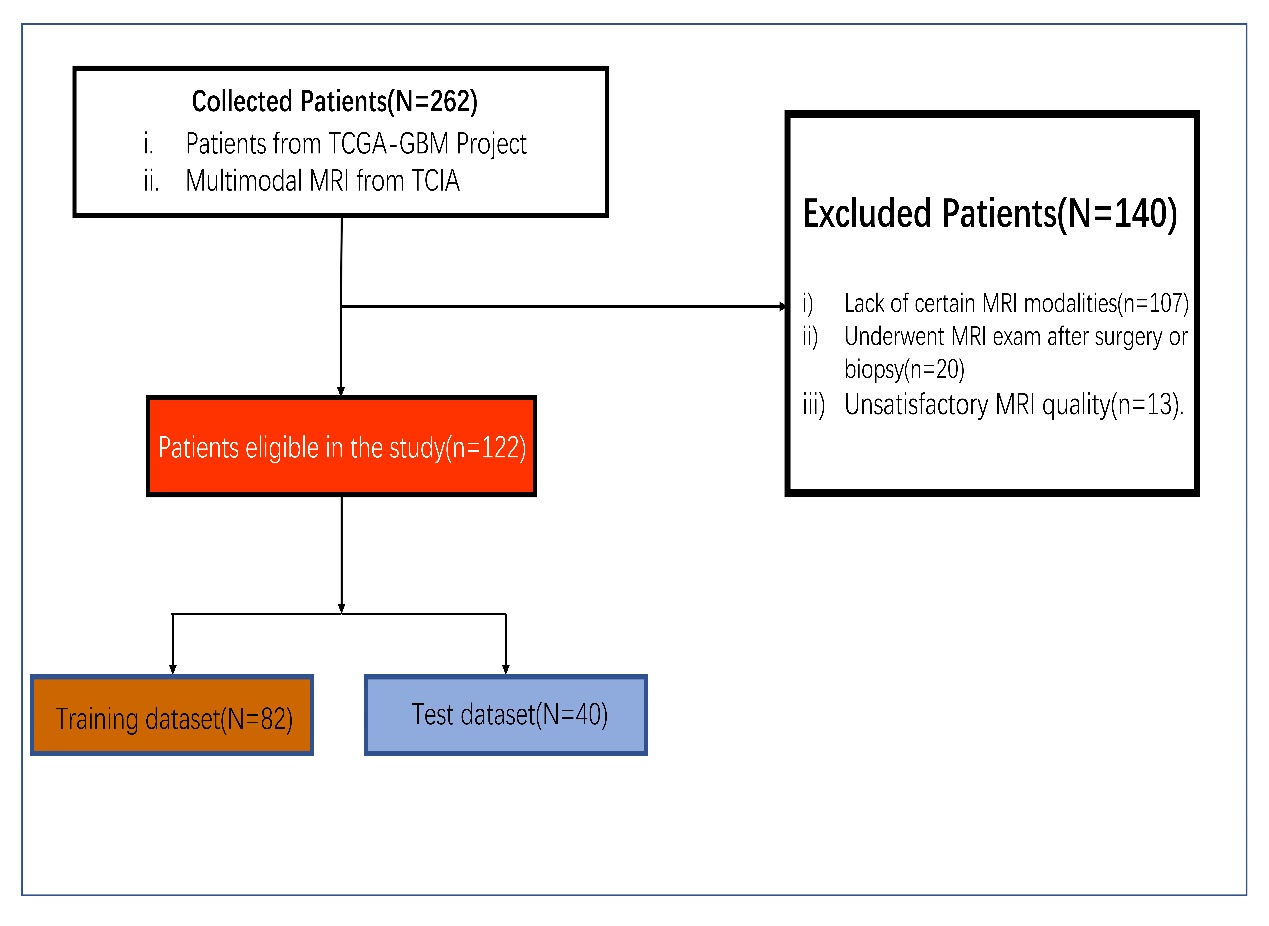
**

**Figure S1. Flow diagram for TCIA patient selection and the distributions of Training and Test groups.**

**Supplement S2. Inclusion and Exclusion Criteria of Local dataset**

**According to the inclusion criteria presented (Fig. S2), a total of 140 patients from Local hospital were retrospectively included. Then, 110 patients were excluded for the following reasons: i) lack of at least one of the following MRI sequences: T1-weighted gadolinium contrast-enhanced, T1-weighted, T2-weighted, and T2-weighted FLAIR sequences (T1C, T1, T2, FLAIR) (n=27); ii) the MRI sequences were acquired after surgery or biopsy (n=26). iii) the MRI sequences were acquired with severe motion or artifacts that may have induced bias in subsequent analyses (n=13);iv) No postoperative radiotherapy in our hospital(N=20);v)Loss to Follow up(N=24)**

**In total, 30 patients were eligible for this study.**

**
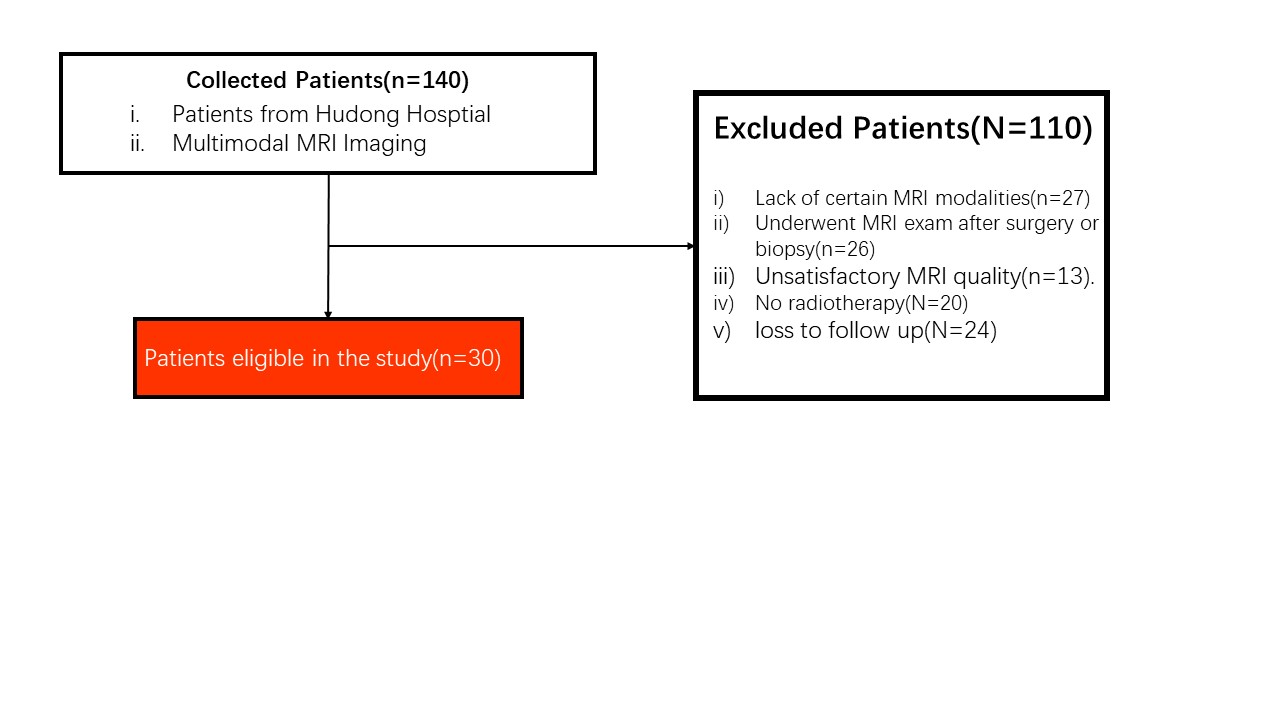
**

**Figure S2. Flow diagram for Local hospital patients selection**

**Supplement S3 Detailed Description of the Radiomics Feature Extraction**

This study extracted 1781 features from each subregion of each MRI sequence，which can be divided into the following groups:

| Group | Name | Numbers |
| --- | --- | --- |
| Group1. | Shape and size Features | 14 |
| Group2. | Intensity Features | 18 |
| Group3. | Textural Features | 75 |
| Group4. | Wavelet Features: | 744 |
| Group5. | Laplace OF Gaussian Features(LOG) | 930 |

**Group definition:**

**Group 1:** Shape and size based Features :(e.g., sphericity, maximal diameter, volume). These

features are based on the 3D representation of a tumor.

**Group 2**: Intensitiy Features: (e.g., mean, standard deviation, kurtosis). These features are based on the histogram of the voxel intensity values of the image.

**Group 3**: Textural features (e.g., entropy, gray-level non-uniformity). These features measure

textural structures.

**Group 4**: Wavelet features (e.g., wavelet energy, wavelet median). To calculate these features, a wavelet filter is first applied to the image before computing Group 2-3 features.

**Group 5**: Laplace of Gaussian (LoG) features (e.g., LoG skewness, LoG uniformity). To calculate

these features, the LoG filter is applied to the image first, which results in highlighted edges. After the LoG filtering, we varied 𝜎 from 0.5mm to 5mm by an increment of 0.5mm for every Group 2-3 features to highlight fine and coarse textures.

The features of group1-3 are shown in the following table. The detailed feature definition can be seen in the website of Pyradiomics:

https://pyradiomics.readthedocs.io/en/latest/features.html

**Group 1.Shape and size Features（14）：**

| Elongation |
| --- |
| Flatness |
| LeastAxisLength |
| MajorAxisLength |
| Maximum2DDiameterColumn |
| Maximum2DDiameterRow |
| Maximum2DDiameterSlice |
| Maximum3DDiameter |
| MeshVolume |
| MinorAxisLength |
| Sphericity |
| SurfaceArea |
| SurfaceVolumeRatio |
| VoxelVolume |

**Group2.Intensitiy Features（18）：**

| 10Percentile |
| --- |
| 90Percentile |
| Energy |
| Entropy |
| InterquartileRange |
| Kurtosis |
| Maximum |
| Mean |
| MeanAbsoluteDeviation |
| Median |
| Minimum |
| Range |
| RobustMeanAbsoluteDeviation |
| RootMeanSquared |
| Skewness |
| TotalEnergy |
| Uniformity |
| Variance |

**Group3.Textural Features（75）：**

**GLCM（24）:**

| glcm_Autocorrelation |
| --- |
| glcm_ClusterProminence |
| glcm_ClusterShade |
| glcm_ClusterTendency |
| glcm_Contrast |
| glcm_Correlation |
| glcm_DifferenceAverage |
| glcm_DifferenceEntropy |
| glcm_DifferenceVariance |
| glcm_Id |
| glcm_Idm |
| glcm_Idmn |
| glcm_Idn |
| glcm_Imc1 |
| glcm_Imc2 |
| glcm_InverseVariance |
| glcm_JointAverage |
| glcm_JointEnergy |
| glcm_JointEntropy |
| glcm_MaximumProbability |
| glcm_MCC |
| glcm_SumAverage |
| glcm_SumEntropy |
| glcm_SumSquares |

**GLDM（14）：**

| gldm_DependenceEntropy |
| --- |
| gldm_DependenceNonUniformity |
| gldm_DependenceNonUniformityNormalized |
| gldm_DependenceVariance |
| gldm_GrayLevelNonUniformity |
| gldm_GrayLevelVariance |
| gldm_HighGrayLevelEmphasis |
| gldm_LargeDependenceEmphasis |
| gldm_LargeDependenceHighGrayLevelEmphasis |
| gldm_LargeDependenceLowGrayLevelEmphasis |
| gldm_LowGrayLevelEmphasis |
| gldm_SmallDependenceEmphasis |
| gldm_SmallDependenceHighGrayLevelEmphasis |
| gldm_SmallDependenceLowGrayLevelEmphasis |

**GLRLM（16）：**

| glrlm_GrayLevelNonUniformity |
| --- |
| glrlm_GrayLevelNonUniformityNormalized |
| glrlm_GrayLevelVariance |
| glrlm_HighGrayLevelRunEmphasis |
| glrlm_LongRunEmphasis |
| glrlm_LongRunHighGrayLevelEmphasis |
| glrlm_LongRunLowGrayLevelEmphasis |
| glrlm_LowGrayLevelRunEmphasis |
| glrlm_RunEntropy |
| glrlm_RunLengthNonUniformity |
| glrlm_RunLengthNonUniformityNormalized |
| glrlm_RunPercentage |
| glrlm_RunVariance |
| glrlm_ShortRunEmphasis |
| glrlm_ShortRunHighGrayLevelEmphasis |
| glrlm_ShortRunLowGrayLevelEmphasis |

**GLSZM（16）:**

| glszm_GrayLevelNonUniformity |
| --- |
| glszm_GrayLevelNonUniformityNormalized |
| glszm_GrayLevelVariance |
| glszm_HighGrayLevelZoneEmphasis |
| glszm_LargeAreaEmphasis |
| glszm_LargeAreaHighGrayLevelEmphasis |
| glszm_LargeAreaLowGrayLevelEmphasis |
| glszm_LowGrayLevelZoneEmphasis |
| glszm_SizeZoneNonUniformity |
| glszm_SizeZoneNonUniformityNormalized |
| glszm_SmallAreaEmphasis |
| glszm_SmallAreaHighGrayLevelEmphasis |
| glszm_SmallAreaLowGrayLevelEmphasis |
| glszm_ZoneEntropy |
| glszm_ZonePercentage |
| glszm_ZoneVariance |

**NGTDM（5）：**

| ngtdm_Busyness |
| --- |
| ngtdm_Coarseness |
| ngtdm_Complexity |
| ngtdm_Contrast |
| ngtdm_Strength |

**Supplement S4. Intraclass correlation coefficient**

**All ROIs were first segmented by GLIRT（GLioma Image SegmenTation and Registration software）and then modified by two neuroradiologists with more than 7 years of MRI experience. In addition, 20 patients were randomly selected from all data sets for reproducibility analysis to validate the consistency of ROIs. Intra-class correlation coefficient (ICC) of 28496 features from different ROI groups were all calculated. In order to maximize the robustness of the research, 0.9 was taken as the relatively high threshold. Furthermore, none of the unqualified features were included in the 8 features selected by Boruta algorithm (ICC values were all higher than 0.9).**

**
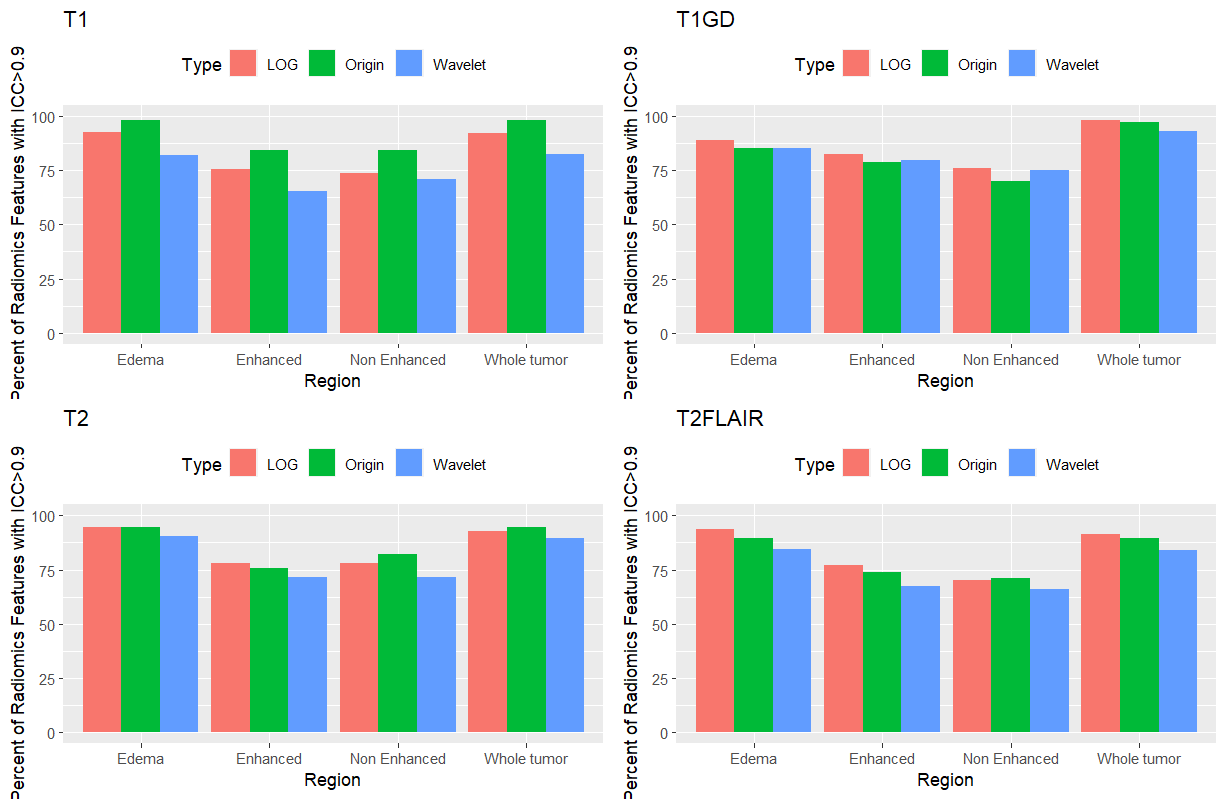
**

**Figure S3. Bar plot of percent radiomics features extracted from various Glioblastoma regions（such as i) edema, ii) enhanced, iii) no-enhanced, and iv)whole tumor ）of T1WI, CE-T1WI, T2, T2FLAIR images with ICC>= 0.9. Wavelet=Features obtained by wavelet fliter；LoG = Features obtained by Laplacian of Gaussian fliter**
